# Care of Older Adults With Complex Needs: Emerging Strategies in the Veterans Health Administration

**DOI:** 10.1093/geroni/igaf122.318

**Published:** 2025-12-31

**Authors:** Maresi Berry-Stoelzle

## Abstract

The Veterans Health Administration (VHA) is unique in its mandate and mission to provide long-term care to eligible Veterans. To meet older Veterans’ increasingly complex needs, VHA has invested in a continuum of residential and community care programs, as well as support for caregiving teams that include paid and unpaid caregivers. This symposium examines promising avenues in VHA for supporting team-based, person-centered long-term care options for growing numbers of older Veterans with complex care needs, many of whom live in rural areas or face other barriers to care access. First, we explore approaches to ensuring Veterans’ access to home health services. Davila et al. describe a successful pilot program to provide VHA-delivered home health (vs. contracted services) to reach high-need rural and other Veterans who may otherwise go unserved. Next, Ding et al. discuss how VHA teams strategically build trust with Veterans and families to encourage uptake of aide services among Veterans who would benefit from these services. We then consider the role of family caregivers. Ngo et al. describe the need to strengthen education and outreach to ensure family caregivers have the information needed to access VHA and community resources, while Quach et al. describe VHA primary care nurses’ experiences coordinating complex care with family caregivers. Finally, Berry-Stoelzle describes how effective billing practices can enhance access to advanced care planning among rural and other hard-to-reach Veterans. Together, this research highlights innovative avenues to improve reach and quality of long-term care for those who need it most.

